# MiR-105-3p acts as an oncogene to promote the proliferation and metastasis of breast cancer cells by targeting GOLIM4

**DOI:** 10.1186/s12885-021-07909-2

**Published:** 2021-03-15

**Authors:** Bo Lin, Chunhua Liu, Enyi Shi, Qiu Jin, Wenhui Zhao, Juan Wang, Runyuan Ji

**Affiliations:** 1 Department of Pathology, Huai’an Key Laboratory of Gastric Cancer, Jiangsu College of Nursing, No. 9 Keji Road, Huai’an, Jiangsu 223001 P.R. China; 2 Office of Educational Administration, Jiangxi University of Traditional Chinese Medicine, Nanchang, 330004 Jiangxi China; 3 Department of Pathology, Suzhou Vocational Health College, Suzhou, 215009 Jiangsu China

**Keywords:** miR-105-3p, Breast cancer, GOLIM4, Proliferation, Migration

## Abstract

**Background:**

Dysregulated miRNAs are involved in carcinogenesis of the breast and may be used as prognostic biomarkers and therapeutic targets during the cancer process. The purpose of this study was to explore the effect of miR-105-3p on the tumourigenicity of breast cancer and its underlying molecular mechanisms.

**Methods:**

Reverse transcription-quantitative polymerase chain reaction (RT-qPCR) was applied to detect the expression of miR-105-3p in breast cancer tissues and cell lines. The impacts of miR-105-3p on the proliferation, migration, invasion and apoptosis of human breast cancer cells (MCF-7 and ZR-75-30) were evaluated by CCK-8 assays, Transwell chamber assays, TUNEL assays and western blot analyses. In addition, bioinformatics and luciferase reporter assays were used to determine the target genes of miR-105-3p.

**Results:**

The expression of miR-105-3p was elevated in breast cancer tissues and increased with tumour severity. Downregulation of miR-105-3p could inhibit cell proliferation, suppress cell migration/invasion, and promote cell apoptosis in MCF-7 and ZR-75-30 cells. Furthermore, Golgi integral membrane protein 4 (GOLIM4) was identified as the direct target gene of miR-105-3p by bioinformatics and luciferase reporter assays. In addition, silencing GOLIM4 restored the anti-breast cancer effects induced by miR-105-3p downregulation.

**Conclusions:**

MiR-105-3p acts as an oncogene to promote the proliferation and metastasis of breast cancer cells by targeting GOLIM4, which provides a new target for the prevention and treatment of breast cancer.

**Supplementary Information:**

The online version contains supplementary material available at 10.1186/s12885-021-07909-2.

## Background

Breast cancer, the most common female malignant tumour worldwide, has the second highest mortality rate among all female malignancies, and the incidence is increasing at a rate of 3% per year in China [1–3]. Major advances have been achieved in the early diagnosis and molecular-targeted therapy of breast cancer due to the development of modern molecular diagnostic technology [4]. The high postoperative recurrence and low postoperative survival rate of patients with advanced breast cancer remain an issue. Therefore, further research should focus on the molecular mechanism underlying the occurrence and development of breast cancer to find new therapeutic targets for breast cancer.

As important single-stranded noncoding RNAs that are 18–25 nucleotides in length, miRNAs have gained much attention for their associations with the growth and invasion of tumours, including breast cancer and stomach cancer [5, 6]. MiRNAs mainly regulate gene expression by binding to the 3′ untranslated region of messenger RNAs during the post-transcriptional stage [7]. Notably, more than half of miRNAs serve as oncogenes or tumour suppressors depending on their targeted mRNAs and are involved in many biological processes in cancer, including cell proliferation, cell cycle, apoptosis, invasion, metastasis, glucose and lipid metabolism, and immune responses [8]. Given that different expression levels of miRNAs have been identified in all stages of breast cancer, miRNAs have become early diagnostic biomarkers and potential therapeutic targets for breast cancer [9]. Bahena et al. found that overexpression of miR-10b in breast cancer cells could inhibit PTEN expression, promote epithelial-mesenchymal transition (EMT), and upregulate stem cell markers expression, thereby promoting breast cancer invasion and metastasis [10]. Damiano and his colleagues found that the downregulation of miR-200 in breast cancer could account for EMT and stem-like features of breast cancer by targeting ZEB1 [11]. All these studies revealed the important roles of miRNAs in breast cancer.

MiR-105-3p is a highly conserved miRNA among humans, cattle, horses and many other species, indicating its multiple potential biological effects. Recent studies have revealed that miR-105-3p is closely related to the occurrence and development of tumours, including ovarian cancer, prostate cancer, colon cancer and hepatocellular carcinoma [12, 13]. Moreover, miR-105-3p could act as an oncogene that affects multiple biological behaviours of tumour growth by regulating the expression of different proteins. However, little information about the expression pattern and biological function of miR-105-3p in breast cancer is known thus far. In this study, we systematically assessed the expression pattern of miR-105-3p in breast cancer tissues and various breast cancer cell lines. We found that the expression level of miR-105-3p was obviously elevated in breast cancer tissues and breast cancer cell lines. The in vitro experiments showed that downregulation of miR-105-3p repressed cell proliferation, migration and invasion in MCF-7 and ZR-75-30 cells, indicating that it was an oncogene in breast cancer. In addition, our study confirmed that miR-105-3p could directly bind to the 3’UTR of GOLIM4 and downregulate the expression of GOLIM4 in MCF-7 and ZR-75-30 cells. All of these results indicated that miR-105-3p played a tumour promoter role in breast cancer.

## Methods

### Cell culture and transfection

Human breast cancer cell lines in this study, including MCF-7 and ZR-75-30, were purchased from the China Infrastructure of Cell Line Resource and cultured in RPMI 1640 medium containing 10% foetal bovine serum (FBS), 100 mg/ml penicillin and streptomycin at 37 °C in 5% CO_2_. Hsa-miR-105-3p inhibitor and its corresponding negative control (NC inhibitor) were synthesized by Sangon Biotech (Shanghai Co., Ltd.) and transfected into the MCF-7 and ZR-75-30 cell lines by Lipofectamine 2000 (Thermo Fisher Scientific, Inc.; USA) according to the manufacturer’s instruction. For silencing of GOLIM4 in MCF-7 and ZR-75-30 cell lines, shRNA targeting GOLIM4 was synthesized by Sangon Biotech (Shanghai, Co., Ltd.) and transfected into these MCF-7 and ZR-75-30 cell lines by Lipofectamine 2000. The successfully transfected cell lines were further studied in the following experiments.

### RT-qPCR assay

Cancer tissues and paired adjacent tissues of 80 patients with breast cancer were collected from our hospital. The MagMAX™ RNA isolation kit and VetMAX™-Plus One-Step RT-PCR Kit (USA; Thermo Fisher Scientific, Inc.) were applied to isolate total RNA and miRNAs according to the instructions. After total RNA was extracted from tumour tissues, a TaqMan microRNA assay (Applied Biosystems; Thermo Fisher Scientific, Inc.) was carried out to measure the expression of miR-105-3p. The reagent components in the reaction system were as follows: 20× TaqMan miRNA assay (1 μL), 2× TaqMan Universal PCR Master Mix (10 μL; USA; Thermo Fisher Scientific, Inc.), cDNA (1.33 μL), forward primer (1 μL) and reverse primer (1 μL) and double distilled water (5.67 μL). RT-qPCR was performed on the ABI 7500 Real-Time PCR System, and the results of the threshold cycle (Ct) were calculated by the 2^−ΔΔCt^ method after normalization to the endogenous control U6 snRNA (forward primer: 5′-ATTGGAACGATACAGAGAAGATT-3′; reverse primer: 5′-GGAACGCTTCACGAATTTG-3′). The expression level of GOLIM4 was detected with the methods described above in breast cancer tissue and cell lines. MiR-105-3p: Forward primer: 5′- CCACGGACGTTTGAGCAT − 3′; Reverse primer: 5′-TATGGTTGTTCACGACTCCTTCAC-3′. GOLIM4: Forward primer: 5′-CAGAGCCAATCCAACAAG-3′; Reverse primer: 5′- ATTGCCGACTCCACGACAC-3′. GAPDH: Forward primer: 5′-TGACTTCAACAGCGACACCCA-3′; Reverse primer: 5′-CACCCTGTTGCTGTAGCCAAA-3′.

### Colony formation assay

ZR-75-30 and MCF-7 cells were cultured to the logarithmic growth phase, and then, the cells were collected and suspended. The concentration of the suspended cells was adjusted to 1 × 10^4^ cell/mL, and a total of 5 × 10^4^ cells were inoculated into 10 cm dishes. The cells were incubated for 2 weeks, and then, methanol was added to fix the formed cell colonies. Subsequently, the cells were stained with crystal violet dye. The number of colonies containing more than 50 cells was counted.

### CCK-8 assay

Cell Counting Kit-8 (Dojindo, Inc.; Japan) was used to detect the proliferative capacity of the transfected ZR-75-30 and MCF-7 cells according to the instruments. Briefly, a total of 5 × 10^3^ cells were seeded in 96-well plates with 10 μL CCK-8 solution. The cells were incubated for 24, 48 and 72 h, and then, the absorbance was measured at 490 nm with a microplate reader.

### Scratch test

ZR-75-30 and MCF-7 cells were transfected with miR-105-3p inhibitor and cultured to the logarithmic growth phase. The bottom of the 6-well plate was rowed with five straight lines with 0.5 cm intervals. After the cells were suspended and adjusted to 1 × 10^5^ cells/mL, 2 mL of cell suspension was added to the well and cultured for 24 h. Subsequently, a scratch perpendicular to the baselines above was made with a 10-μl pipet. The cells were cultured for 48 h with serum-free medium, and wound closure (%) was finally calculated.

### Terminal deoxynucleotidyl transferase (TdT)-mediated dUTP nick end labelling (TUNEL) assay

A TUNEL assay was performed to detect the apoptosis of transfected cells using a One Step TUNEL Apoptosis Assay Kit (Beyotime Biotechnology Co., Ltd.; China) following the manufacturer’s instructions. Briefly, a total of 5 × 10^3^ cells were seeded in 96-well plates for 48 h of culture. Then, the cells were fixed with 4% paraformaldehyde for 30 min at room temperature. After two washes with PBS, 0.1% Triton X-100 was added to permeabilize the cell membrane. The cells were successively treated with a TUNEL reaction mixture, converter-POD and DAPI substrate. The cell images were then obtained under a fluorescence microscope (Olympus, Tokyo, Japan).

### Transwell chamber assay

Transwell chamber assays were applied to detect the invasion of ZR-75-30 and MCF-7 cells after transfection with NC or miR-105-3p inhibitor. FBS-free culture medium and culture medium containing 10% FBS were added to the upper and lower chambers, respectively. The transfected cells were cultured in FBS-free culture medium for 12 h, and then, 2 × 10^4^ cells were seeded in the upper chamber with 10 mg/mL Matrigel. After culture for 48 h, the top of the filter was carefully wiped with cotton swabs to remove the remaining cells. Cells that migrated through the membrane were fixed with 95% methanol, stained with 0.5% crystal violet and finally counted with a microscope (magnification, × 200; Olympus Corporation, Tokyo, Japan).

### Western blot assay

After the transfected cells were cultured for 72 h, the cells were washed with PBS three times and then centrifuged to obtain cell pellets. The ProteoPrep® Total Extraction Sample Kit was used to extract total protein according to the instructions followed by the detection of the protein concentration using a BCA assay kit (USA; Thermo Fisher Scientific, Inc.). Equal quantities of protein were separated by 12% SDS-PAGE and subsequently transferred to polyvinylidene difluoride (PVDF) membranes (Thermo Fisher Scientific, Inc.; USA). After the membranes were blocked with 5% milk in PBST for 1 h at room temperature, it was incubated with proper dilutions of primary antibodies for 1 h at 37 °C; the antibodies included anti-BAX, anti-Bcl-2, anti-cleaved caspase-3, anti-cleaved caspase-9, anti-ICAM-1 and anti-VCAM-1 antibodies. β-actin was chosen as an internal control in the assay. After three times of wash with TBST, HRP-labelled secondary antibodies corresponding to primary antibodies were used to probe the expression of the target proteins in the membranes. The protein bands were visualized using a Novex™ ECL Chemiluminescent Substrate Reagent Kit (Thermo Fisher Scientific, Inc., USA). Densitometric quantification of protein bands was conducted using ImageJ Pro Plus software and then normalized to β-actin.

### Luciferase reporter assay

For analysis of the relationship between miR-105-3p and GOLIM4, ZR-75-30 and MCF-7 cells were cotransfected with miR-NC or miR-105-3p and the psiCHECK2 vector containing the wild-type or mutant GOLIM4 fragment (psiCHECK2- GOLIM4–3’UTR WT or psiCHECK2- GOLIM4–3’UTR MUT) with Lipofectamine 2000 (Thermo Fisher Scientific, Inc.; USA). After culture for 48 h and three washes, the cells were lysed with harvest buffer for 10 min at 0 °C. A mixture of ATP buffer and luciferin buffer (1:3.6) was added to the cell lysate, and the absorbance was detected.

### Statistical analysis

The measurement data that conformed to a normal distribution are shown as the mean ± standard deviation. The difference between two groups was analysed by Student’s t-test. One-way ANOVA was used to analyse the differences among groups followed by Bonferroni post hoc analysis. The 80 patients were divided into the high and low miR-105-3p expression groups according to the median miRNA expression, and then, life table methods were used to analyse the difference in survival data between the two groups, after which the differences in survival time were tested by the *Mantel-Cox log-rank method*. All the data were analysed by SPSS software, version 20 (SPSS, Inc., Chicago, IL, USA). A *P* value < 0.05 was considered to indicate a significant effect.

## Results

### The expression levels of miR-105-3p in breast cancer tissues and cell lines

**Fig. 1:**
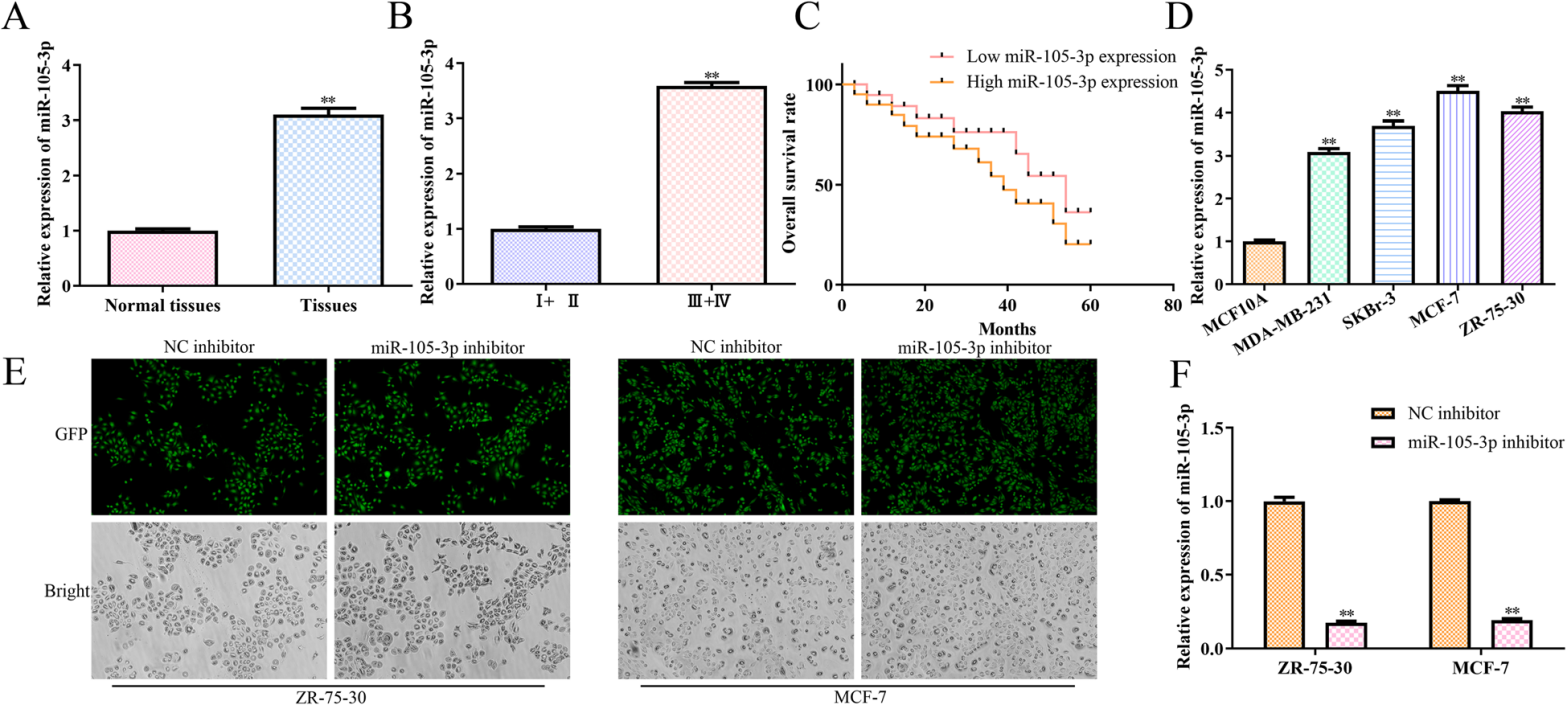
The expression of miR-105-3p in breast cancer tissues and cell lines. **a** The expression of miR-105-3p in breast cancer and adjacent noncancerous tissues. **b** The expression of miR-105-3p in breast cancer tissues with different tumour stages. **c** Kaplan–Meier survival curve analyses among breast cancer patients with different expression levels of miR-105-3p. **d** The expression level of miR-105-3p in the breast cancer cell lines MCF10A, MDA-MB-231, SKBr-3, MCF-7 and ZR-75-30. **e** Transfection efficiency of the miR-105-3p miRNA inhibitor and NC inhibitor in MCF-7 and ZR-75-30 cells. **f** The expression level of miR-105-3p in the MCF-7 and ZR-75-30 cells transfected with miR-105-3p miRNA inhibitor and NC inhibitor. Asterisks indicate significant differences from the control (**P < 0.01, Student’s *t*-test, compared to normal tissues, breast cancer of stage I + II, the MCF-10A group or the NC inhibitor group)

The expression of miR-105-3p in breast cancer and adjacent noncancerous tissues of 80 breast cancer patients with different stages was detected with RT-qPCR. Compared with that of the adjacent noncancerous tissues, miR-105-3p expression was elevated in the tumour tissues (Fig. 1a). Of note, the expression levels of miR-105-3p were higher in the tumour tissues at stages III and IV than in the tumour tissues at stages I and II (Fig. 1b). In addition, the survival curve analysis indicated that the survival time of patients with high expression levels of miR-105-3p was shorter than that of patients with low expression levels (Fig. 1c, *P <* 0.05). To choose suitable breast cancer cell lines to evaluate the biological function of miR-105-3p, we determined the expression levels of miR-105-3p in several breast cancer cell lines, including MCF10A, MDA-MB-231, SKBr-3, MCF-7 and ZR-75-30, by RT-qPCR. We found that the expression levels of miR-105-3p in the MCF-7 and ZR-75-30 cell lines were the highest among the five cell lines (Fig. 1d). Thus, we chose these two cell lines as models for further research. To identify the role of miR-105-3p in the regulation of breast cancer progression, we first transfected hsa-miR-105-3p inhibitor and its corresponding negative control (NC inhibitor) into the indicated cells. Cellular immunofluorescence showed that the transfected cells contained green fluorescence (Fig. 1e). Furthermore, RT-qPCR analysis showed that the expression level of miR-105-3p was successfully downregulated by the miR-105-3p inhibitor, which suggested that the miR-105-3p inhibitor could be used in the following experiments (Fig. 1f).

### Downregulation of miR-105-3p inhibits cell proliferation and promotes apoptosis of MCF-7 and ZR-75-30 cells

**Fig. 2:**
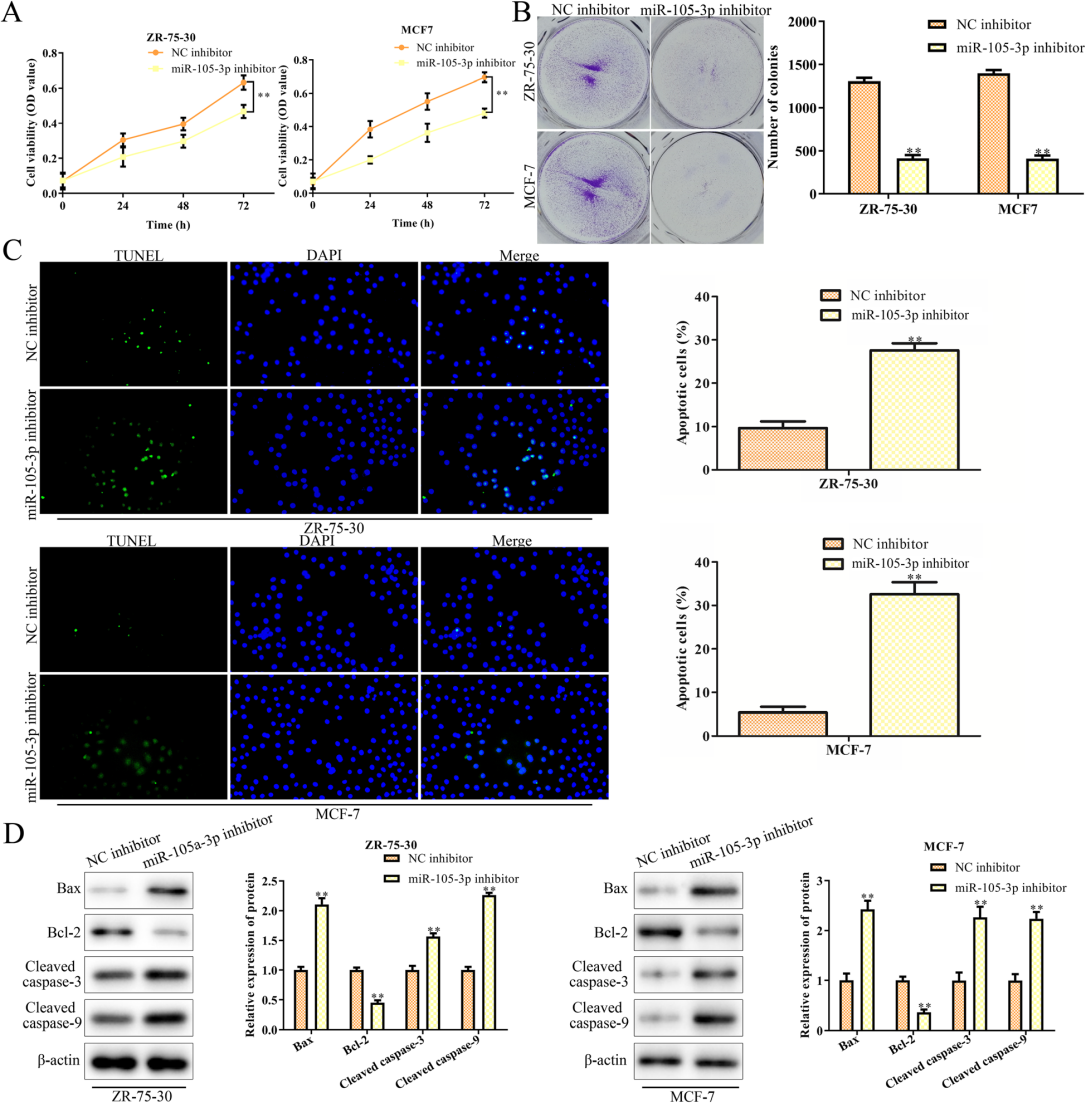
Downregulation of miR-105-3p inhibits cell proliferation and promotes apoptosis of MCF-7 and ZR-75-30 cells. **a** MCF7 and ZR-75-30 cells were transfected with either NC inhibitor or miR-105-3p inhibitor for 72 h. **a** CCK-8 assay of the viability of MCF-7 and ZR-75-30 cells. **b** Scratch test. **c** TUNEL assay. **d** Western blot analysis of the related proteins involved in cell apoptosis, including Bax, Bcl-2, cleaved caspase-3, and cleaved caspase-9. Asterisks indicate significant differences from the control (**P < 0.01, Student’s t-test, compared to the NC inhibitor)

To evaluate the effect of miR-105-3p in MCF-7 and ZR-75-30 cells, we transfected the two cell lines with miR-105-3p inhibitor or NC inhibitor. We found that downregulation of miR-105-3p could suppress cell proliferation according to the results of CCK-8 and colony formation assays (Fig. 2a and b). In addition, a TUNEL assay was performed to measure cell apoptosis. The results showed that the numbers of apoptotic MCF-7 and ZR-75-30 cells transfected with the miR-105-3p inhibitor were increased compared to those transfected with the NC inhibitor (Fig. 2c). Furthermore, the western blot results revealed that the miR-105-3p inhibitor increased the expression levels of Bax, cleaved caspase-3 and cleaved caspase-9 while decreasing the expression levels of Bcl2 in MCF-7 and ZR-75-30 cells (Fig. 2d). These data suggested that knockdown of miR-105-3p inhibited breast cancer cell proliferation and triggered cell apoptosis.

### Downregulation of miR-105-3p suppresses the migration and invasion of MCF-7 and ZR-75-30 cells

**Fig. 3:**
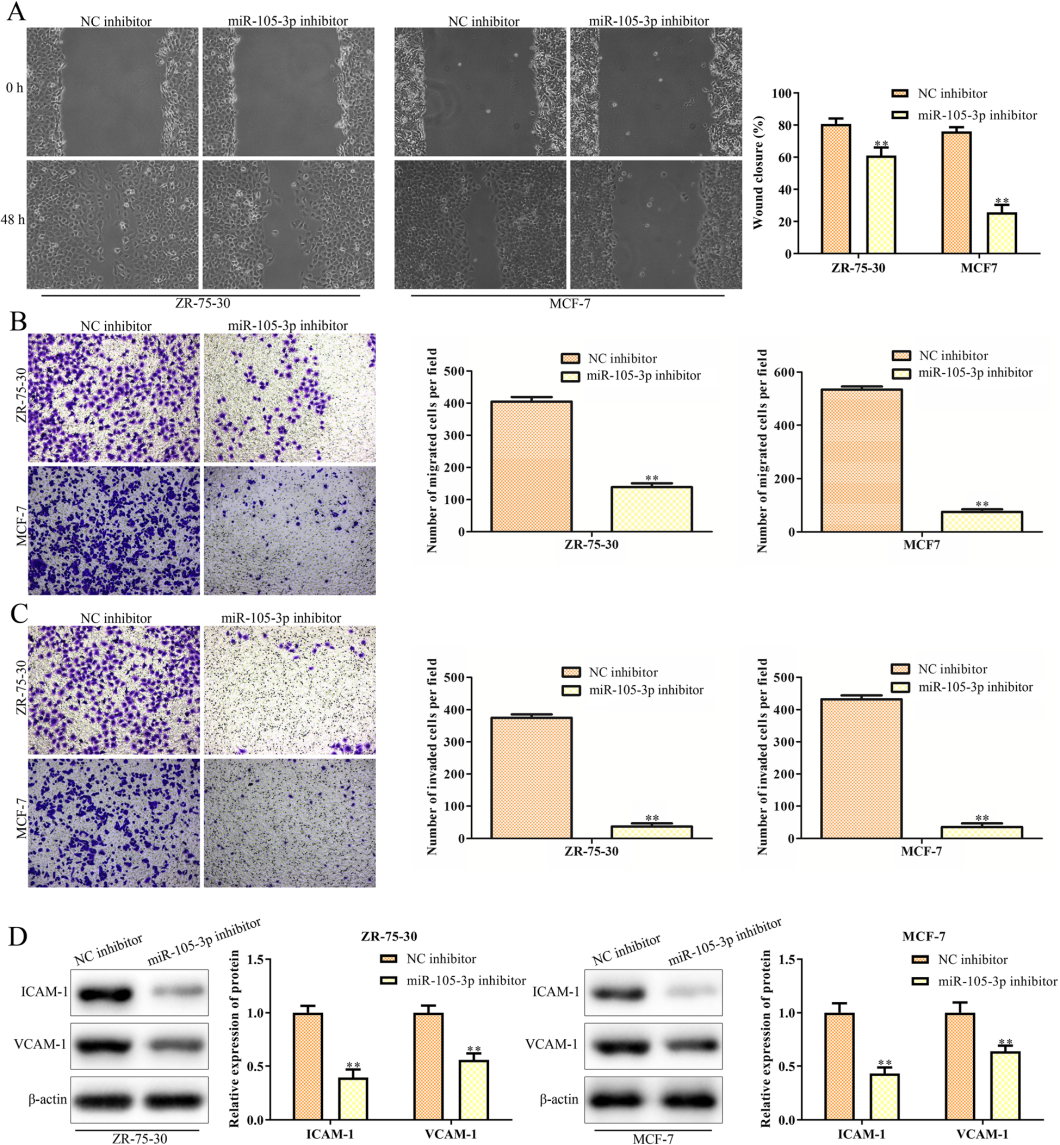
Downregulation of miR-105-3p suppresses the migration and invasion of MCF-7 and ZR-75-30 cells. **a** Scratch test for cell migration. **b**, **c** Cell migration and invasion were detected by Transwell chamber assays. **d** Western blot analysis for ICAM-1 and VCAM-1, which are involved in cell migration and invasion. Asterisks indicate significant differences from the control (**P < 0.01, Student’s t-test, compared to the NC inhibitor group)

To investigate the effects of miR-105-3p on the migration and invasion of MCF-7 and ZR-75-30 cells, we performed scratch tests and Transwell chamber assays in vitro. The results of the scratch test showed that downregulation of miR-105-3p could inhibit the wound closure after 24 h of scratch injury in MCF-7 and ZR-75-30 cells (Fig. 3a). Moreover, Transwell chamber assays revealed that the miR-105-3p inhibitor decreased the numbers of migrated and invaded cells compared with the NC inhibitor (Fig. 3b-c). Given the important function of ICAM-1 and VCAM1–1 in the regulation of cell migration and invasion, we detected the expression levels of these two proteins in the MCF-7 and ZR-75-30 cells with knockdown of miR-105-3p. The result of western blot showed that downregulation of miR-105-3p suppressed the expression of ICAM-1 and VCAM-1 (Fig. 3d). All these data indicated that miR-105-3p was a pivotal regulator involved in breast cancer cell migration and invasion.

### MiR-105-3p modulates GOLIM4 expression by directly targeting its 3’UTR

**Fig. 4:**
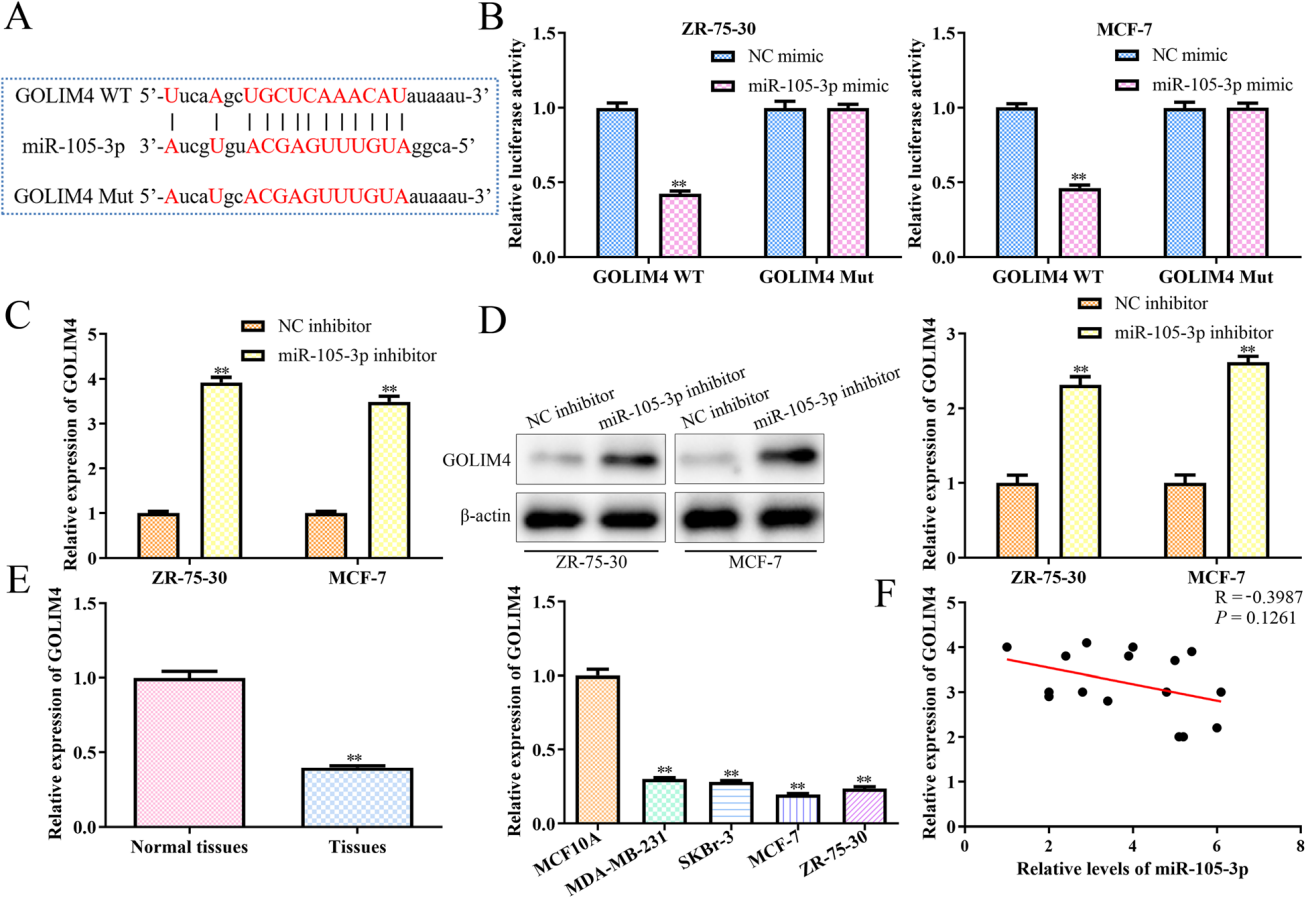
MiR-105-3p modulates GOLIM4 expression in MCF-7 and ZR-75-30 cells by directly targeting its 3’UTR. **a** The predicted miR-105-3p target sequence in the 3’UTR of GOLIM4 and a Mut sequence containing altered nucleotides in the 3’UTR of GOLIM4. **b** Luciferase reporter assays for luciferase activities in the MCF-7 and ZR-75-30 cells synchronously transfected with miR-105-3p mimic or NC mimic together with psiCHECK2- GOLIM4–3’UTR WT or psiCHECK2- GOLIM4–3’UTR MUT. **c**, **d** RT-qPCR and western blot analysis of GOLIM4 in MCF-7 and ZR-75-30 cells after transfection with the miR-105-3p inhibitor. Asterisks indicate significant differences from the control. **e** The expression of GOLIM4 in tumour and control tissues as well as breast cancer cell lines. **f** Correlation analysis of the expression of GOLIM4 and miR-105-3p in breast cancer tissues. (**P < 0.01, Student’s t-test, compared to the NC mimic group or NC inhibitor group)

As predicted by TargetScan and miRanda, GOLIM4 was identified as a potential target gene of miR-105-3p (Fig. 4a). To identify the direct inhibitory effects of miR-105-3p on the expression of GOLIM4, we performed a luciferase reporter assay and found that overexpression of miR-105-3p obviously increased the luciferase activities of the GOLIM4–3’UTR WT but exhibited modest effects on the GOLIM4–3’UTR MUT (Fig. 4b). Consistently, knockdown of miR-105-3p in MCF-7 and ZR-75-30 cells increased the expression of GOLIM4 at both the transcriptional and translational levels (Fig. 4c, d). In addition, the expression levels of GOLIM4 in breast cancer tissues and cell lines were significantly decreased (Fig. 4e). Correlation regression analysis showed a negative correlation between the expression levels of miR-105-3p and GOLIM4 in breast cancer tissues (R = -0.39, *P* = 0.13, Fig. 4f).

### Silencing of GOLIM4 partially disrupts the miR-105-3p inhibitor-induced anti-breast cancer effects

**Fig. 5:**
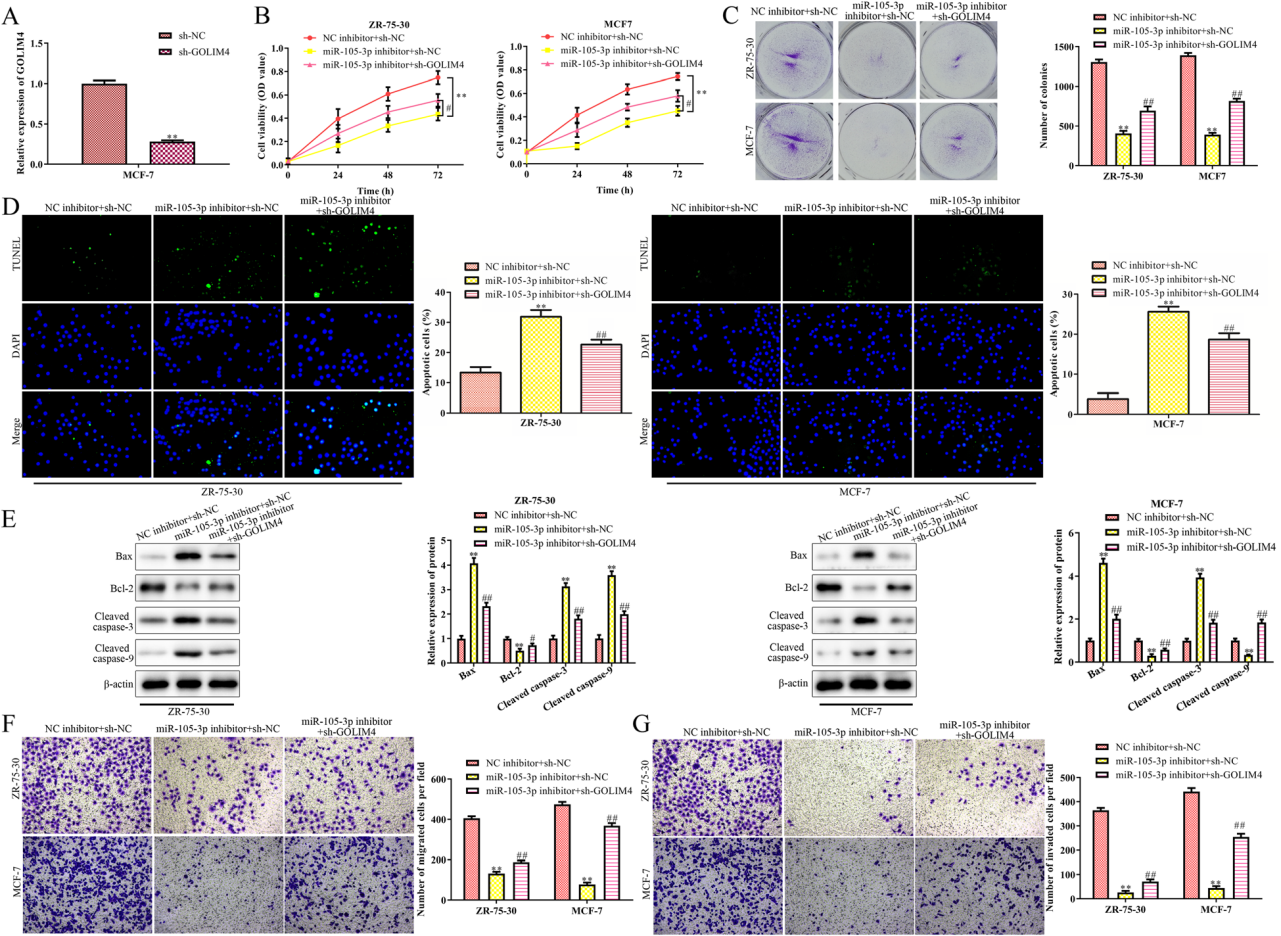
Silencing of GOLIM4 restores cell proliferation and migration and inhibits apoptosis induced by the downregulation of miR-105-3p. Either sh-NC or sh-GOLIM4 was transfected into miR-105-3p-downregulated MCF7 and ZR-75-30 cells, and the cells cotransfected with NC inhibitor and sh-NC were chosen as controls. **a** RT-qPCR analysis of the expression level of GOLIM4 in MCF-7 and ZR-75-30 cells after transfection with sh-NC or sh-GOLIM4. (B, C) CCK-8 and colony formation assays for cell viability and proliferation. **d** TUNEL assay for cell apoptosis. **e** Western blot analysis for related proteins that were involved in cell apoptosis. **e** Transwell chamber assay for cell migration and invasion. Asterisks indicate significant differences from the control (**P < 0.01, compared to the sh-NC and NC inhibitor+ sh-NC group. ^##^*P* < 0.01, compared to miR-105-3p miRNA inhibitor + sh-GOLIM4)

To confirm that miR-105-3p could act as an oncogene to promote the proliferation and metastasis of breast cancer cells by silencing GOLIM4, we silenced the expression of GOLIM4 in the MCF-7 and ZR-75-30 cells transfected with the miR-105-3p inhibitor with sh-GOLIM4. As shown in Fig. 5a, the expression of GOLIM4 in MCF-7 cells was successfully downregulated by transfection with sh-GOLIM4. Functionally, the miR-105-3p inhibitor-induced inhibitory effects on cell proliferation and migration were partially impaired when GOLIM4 expression was silenced in these two cell lines (Fig. 5b and c). In addition, the silencing of GOLIM4 inhibited the apoptosis induced by the miR-105-3p inhibitor. The results of TUNEL and western blot assays showed that sh-GOLIM4 decreased the number of TUNEL-positive cells and decreased the expression of apoptotic proteins, including Bax, cleaved caspase-3 and cleaved caspase-9, in the MCF-7 and ZR-75-30 cells transfected with the miR-105-3p inhibitor compared with the cells cotransfected with sh-NC (Fig. 5d and e). In addition, silencing GOLIM increased the migration and invasion of the MCF-7 and ZR-75-30 cells transfected with the miR-105-3p inhibitor (Fig. 5f and g). These data collectively indicated that miR-105-3p could promote the proliferation and metastasis of breast cancer cells by silencing GOLIM4.

## Discussion

The incidence of breast cancer incidence has sharply increased, and breast cancer is now the second most lethal cancer among women, although major advantages have been made in the diagnosis and treatments of this disease [14]. To date, miRNAs have been proven to be master regulators of tumour progression in various malignancies, including breast cancer [15, 16]. In this study, we showed that elevated expression of miR-105-3p could be found in breast cancer tissues and increased with increasing tumour severity. Downregulation of miR-105-3p could inhibit cell proliferation, suppress cell migration and invasion and promote cell apoptosis in MCF-7 and ZR-75-30 cells. All of these results indicated that miR-105-3p acts as an oncogene in breast cancer. Furthermore, this research identified GOLIM4 as a downstream gene for miR-105-3p since silencing of GOLIM4 could restore the cell proliferation and migration and inhibit the apoptosis induced by the downregulation of miR-105-3p in MCF-7 and ZR-75-30 cells.

The discovery of miRNAs provides new directions to investigate the pathogenesis of tumours, as well as new strategies for the diagnosis and treatment of tumours. Various studies have shown that tumour-specific miRNAs contribute to precision medicine in malignancies by serving as potential therapeutic targets and early diagnostic indicators [9]. In the field of breast cancer, several miRNAs have been identified and found to be extensively involved in the occurrence and development of various cancers by modulating the expression of key proteins at the post-transcriptional level [9]. For example, miR-10b, miR-200 and miR-21 have been demonstrated to be important miRNAs that could be upregulated in breast cancer. These molecules also serve as oncogenes by targeting PTEN, TGF-β and some other tumour-related proteins [16]. Our study revealed that the expression of miR-105-3p was upregulated in breast cancer tissues and that the level was elevated according to the development of tumours. These results indicated that miR-105-3p may act as a potential prognostic factor for breast cancer.

MiR-105-3p is a well-studied miRNA that is an independent predictor of prognosis and acts as an oncogene in oesophageal cancer [17], triple-negative breast cancer [18], and colorectal cancer [19]. For instance, Gao, R. and colleagues reported that miR-105 was significantly upregulated in oesophageal cancer tissues and cell lines and that overexpression of miR-105 was significantly associated with positive lymph node metastasis, advanced TNM stage, and poor overall survival. In addition, overexpression of miR-105 promoted cell proliferation, migration, and invasion in oesophageal cancer cells [17]. Similarly, Li, H. Y. and colleagues found that miR-105 was upregulated and correlated with poor survival in TNBC patients. MiR-105 was found to activate Wnt/beta-catenin signalling by downregulating SFPR1. In addition, high circulating miR-105 (81%) and miR-93-3p (97%) levels were significantly associated with the TNBC subtype, and high expression of circulating miR-105/93-3p (97%) also showed a strong correlation with the TNBC subtype [18]. In this study, the proliferation, invasion and migration of breast cancer cells were significantly inhibited when miR-105-3p was knocked down in MCF-7 and ZR-75-30 cells. These results were similar to those of previous studies in other kinds of tumours. We further detected cell apoptosis in breast cancer cell lines. As expected, knockdown of miR-105-3p in breast cancer cells promoted the apoptosis of MCF-7 and ZR-75-30 cells. Thus, the expression level of miR-105-3p was correlated with the growth and metastatic potential of breast cancer cells, indicating its essential role in governing breast cancer cell progression.

For the molecular mechanism underlying the regulatory effect of miR-105-3p on breast cancer, it is important to dissect its target gene. Thus, two publicly available miRNA databases, named TargetScan and miRanda, were used, and the results showed that GOLIM4 was the potential target gene of miR-105-3p. GOLIM4, also named GPP130, is a membrane-binding protein in the Golgi apparatus and plays a vital role in transporting proteins between the Golgi apparatus and endosomes [20]. Given that dysfunction of the Golgi and endosomes was involved in the progression of various tumours, GOLIM4 was considered to be a tumour suppressor gene in the carcinogenesis of human head and neck cancer [21, 22]. The increased expression of GOLIM4 could inhibit the proliferation of neck cancer, promote cell apoptosis and induce G1 phase arrest in human head and neck cancer cell lines, such as FaDu and Tca-8113 [21]. The luciferase reporter assay in this study provided evidence for GOLIM4 as a potential target of miR-105-3p. The results revealed that miR-105-3p overexpression suppressed the luciferase activities of the WT 3’UTR of GOLIM4; however, no inhibitory effect on the MUT 3’UTR of GOLIM4 was detected, which indicates that miR-105-3p could directly bind to the 3’UTR of GOLIM4. Most importantly, silencing GOLIM4 reversed the inhibitory effect on the biological characteristics of breast cancer cells induced by miR-105-3p knockdown in these cancer cells. Since GOLIM4 has been identified as a tumour suppressor gene in other kinds of cancers, we confirmed that the elevated expression of miR-105-3p could suppress the expression of GOLIM4 by binding to its 3’UTR in the carcinogenesis of breast cancer. These data provide additional evidence for the idea that miR-105-3p acts as an oncogene to promote the proliferation and metastasis of breast cancer cells by targeting GOLIM4. However, the results of the correlation regression analysis showed that the negative correlation (R = -0.39) between the expression levels of miR-105-3p and GOLIM4 in breast cancer tissues was not significant (*P* = 0.13). These results indicated that other proteins may be involved in the function of miR-105-3p, since the molecular mechanisms in tumour cells are complicated.

In summary, miR-105-3p was upregulated in breast cancer tissue and was correlated with tumour stage. The in vitro experiments verified the importance of miR-105-3p in the tumour invasion process, including the promotion of cell proliferation, the enhancement of cell migration, the facilitation of invasion, and the suppression of cell apoptosis. All these effects of miR-105-3p were partially mediated by its inhibitory effect on the expression of GOLIM4. Our findings provide promising evidence that miR-105-3p is a potential target for the clinical treatment of breast cancer and might predict the prognosis of breast cancer patients.

## Conclusion

Elevated expression level of miR-105-3p was correlated with tumour stage. Downregulation of miR-105-3p repressed cellular proliferation, migration and invasion of MCF-7 and ZR-75-30 cells, which indicates that miR-105-3p acts as an oncogene in breast cancer. Furthermore, miR-105-3p could directly bind to the 3’UTR of GOLIM4 and thus act as a tumour promoter in breast cancer. All these results indicated that miR-105-3p might serve as a potential therapeutic target in the precise treatment of breast cancer, and it could also predict the prognosis of breast cancer patients.

## Supplementary Information

**Additional file 1: Supplemental Figure 1**. Dosage optimization of the miR-105-3p inhibitor in MCF-7 and ZR-75-30 cells. RT-qPCR was performed to detect the expression levels of miR-105-3p and GOLIM4 in MCF-7 and ZR-75-30 cells after transfection with different doses of miR-105-3p inhibitor (25 nM, 50 nM, 100 nM, 200 nM and 400 nM). The RT-qPCR results showed that as the dose of the miR-105-3p inhibitor increased from 25 nM to 100 nM, the expression levels of miR-105-3p decreased gradually, while the expression levels of GOLIM4 increased. In addition, the expression levels of miR-105-3p and GOLIM4 were not remarkably changed among the doses of miR-105-3p inhibitor from 100 nM to 400 nM. Therefore, a dosage of 100 nM was chosen in the following experiments.

## Data Availability

The datasets used and/or analysed during the current study are available from the corresponding author on reasonable request.

## Abbreviations

miR: microRNA
HCC: Hepatocellular carcinoma
TGF-β: Transforming growth factor-β
PTEN: Phosphatase and tensin homologue deleted on chromosome ten
EMT: Epithelial-mesenchymal transition
GOLIM4: Golgi integral membrane protein 4
