# Supplementary figures and images for: MiR-105-3p acts as an oncogene to promote the proliferation and metastasis of breast cancer cells by targeting GOLIM4
Source: BMC Cancer. 2021 Mar 15;21:275. doi: 10.1186/s12885-021-07909-2 (PMC7962220; doi:10.1186/s12885-021-07909-2)

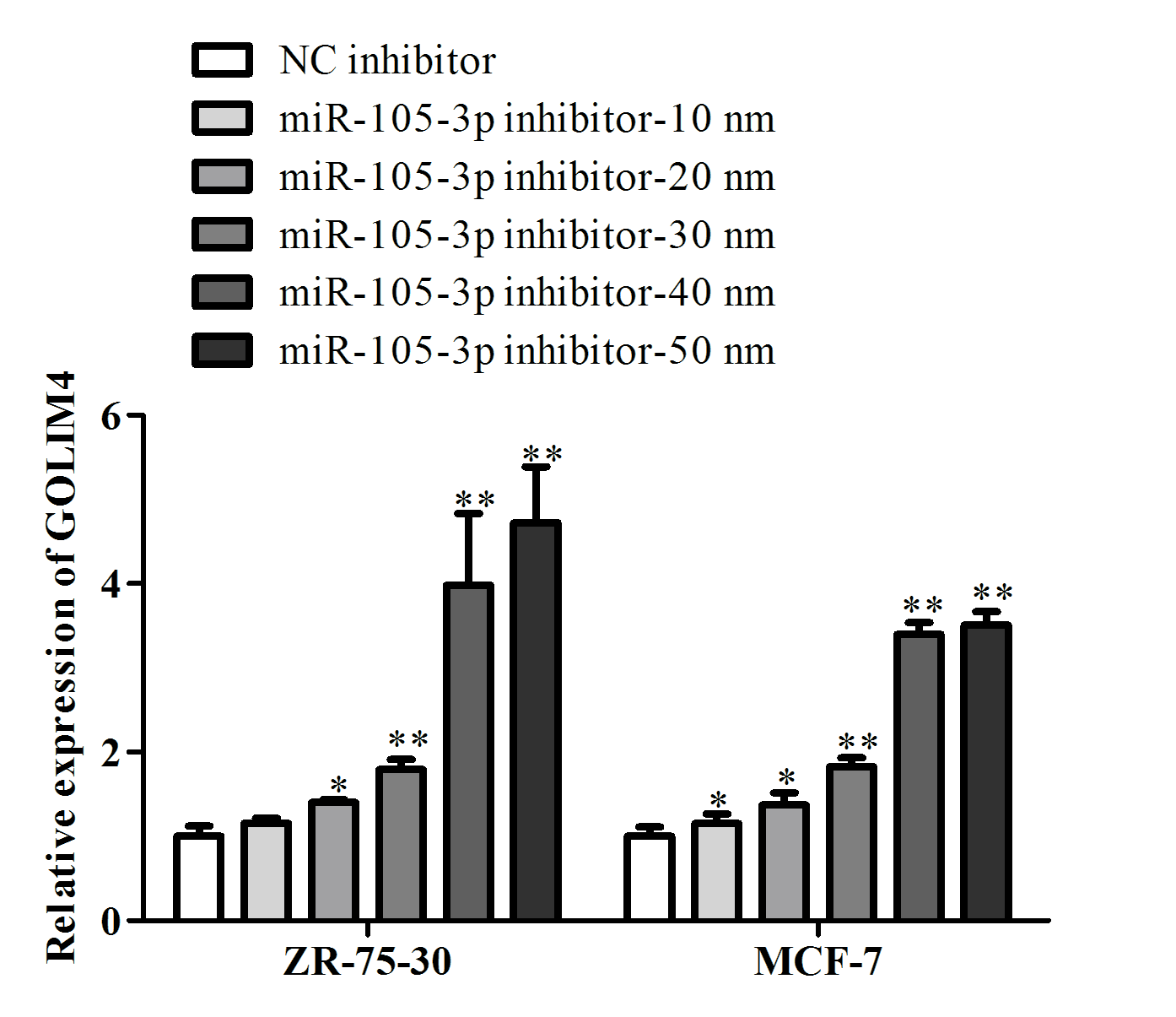

Supplement: Supplementary file 1 — Additional file 1: Supplemental Figure 1. Dosage optimization of the miR-105-3p inhibitor in MCF-7 and ZR-75-30 cells. RT-qPCR was performed to detect the expression levels of miR-105-3p and GOLIM4 in MCF-7 and ZR-75-30 cells after transfection with different doses of miR-105-3p inhibitor (25 nM, 50 nM, 100 nM, 200 nM and 400 nM). The RT-qPCR results showed that as the dose of the miR-105-3p inhibitor increased from 25 nM to 100 nM, the expression levels of miR-105-3p decreased gradually, while the expression levels of GOLIM4 increased. In addition, the expression levels of miR-105-3p and GOLIM4 were not remarkably changed among the doses of miR-105-3p inhibitor from 100 nM to 400 nM. Therefore, a dosage of 100 nM was chosen in the following experiments. [file 12885_2021_7909_MOESM1_ESM.tif]
